# Capsular bag performance of an aspheric hydrophobic IOL after conventional phacoemulsification or femtosecond laser-assisted cataract surgery: 12-month results of a randomized prospective study

**DOI:** 10.1007/s00417-025-06919-1

**Published:** 2025-07-18

**Authors:** Marina Casazza, Sophia Anna Reifeltshammer, Nino Hirnschall, Jascha Wendelstein, Siegfried Mariacher, Peter Laubichler, René Siska, Matthias Bolz

**Affiliations:** 1 Department of Ophthalmology, Medical University Graz, Neue Stiftingtalstraße 6, Graz, 8010 Austria; 2 Department of Ophthalmology and Optometry, Kepler University Hospital, Linz, Austria; 3 Medical Faculty, Johannes Kepler University, Linz, Austria; 4 Department of Ophthalmology, Ludwig-Maximilians-University, Munich, Germany; 5 Private Practice, Landeck and Innsbruck, Austria

**Keywords:** Cataract, Femtolaser assisted cataract surgery, Intraocular lens stability, Intraocular lens

## Abstract

**Purpose:**

To evaluate a new hydrophobic aspheric intraocular lens (IOL) regarding its refractive and position stability after conventional (CCS) or femtosecond laser-assisted cataract surgery (FLACS).

**Methods:**

All patients received the same IOL (CT LUCIA 621P, Carl Zeiss Meditec AG, Germany). Both eyes of each patient were randomized, with one eye assigned to the FLACS group and the other eye to the CCS group. One, 6 and 12 months after surgery, 2 different swept-source optical coherence tomography measurements (IOLMaster 700, Carl Zeiss Meditec AG, Germany and CASIA-2, Tomey GmbH, Japan) were performed as well as subjective refraction, best corrected (BCDVA) and uncorrected visual acuity (UDVA).

**Results:**

A total of 74 eyes of 37 patients were included in this study. Mean postoperative BCDVA for the FLACS and CCS group was − 0.08 ± 0.08 logMAR and 0.08 ± 0.07 logMAR, respectively. Overall, mean absolute error (MAE) and mean error (ME) was 0.48 ± 0.38dpt and 0.42 ± 0.44dpt respectively with no differences between groups (*p* = 0.559 and *p* = 0.786). Overall, tilt magnitude was stable in all groups over the course of 12 months with a mean tilt magnitude of 4.48 ± 2.48° at 12 months. Postoperative patient satisfaction was high with 97.3% of all patients very or fairly satisfied with the outcome.

**Conclusion:**

There was no significant difference between groups regarding postoperative stability and capsular bag performance as well as refractive error over the course of 12 months. The CT LUCIA 621P demonstrated good efficacy, safety and stability. No issues regarding biocompatibility of the IOL material were observed.

## Key messages

*****What was known***:**

- Femtosecond laser assisted surgery is a safe alternative to conventional cataract surgery providing similar outcomes.

*****What was added***:**

- This study evaluated both conventional and femtosecond laser assisted surgery in a bilateral comparison.
- The CT LUCIA 621P (CZM AG, Germany) showed good postoperative stability and refractive results.

## Introduction

Patient satisfaction strongly depends on the post operative refraction, which is mainly influenced by intraocular lens (IOL) alignment in the capsular bag. Different parameters have a significant impact on this alignment, such as the size of the capsular bag, the design of the IOL, the post-operative interaction between the capsular bag and the IOL and the rhexis size and shape [1]. To achieve a standardized rhexis, the usage of a femtosecond laser (femtosecond laser assisted cataract surgery, FLACS) could be used. Although investigated in several studies, there still is no evidence for the benefit of FLACS regarding the post-operative refractive outcome [2]. 

This study aimed to evaluate the capsular bag and refractive stability of the CT LUCIA 621P IOL within the first 12 months after surgery.

## Methods

This prospective randomized, examiner-masked study with bilateral, intraindividual comparison included patients scheduled for cataract surgery at a tertiary care center (Kepler University Hospital, Linz, Austria). Inclusion criteria were necessity of cataract surgery in both eyes at at least 21 years of age. Exclusion criteria were relevant ophthalmic diseases that could influence the capsular bag or visual performance, such as pseudoexfoliation syndrome, history of ocular trauma, severe corneal scarring, and risk factors for an intra-operative floppy iris syndrome. The research and measurements followed the tenets of the Declaration of Helsinki. Written informed consent was obtained from all patients after the possible consequences of the study had been explained, and the study was approved by the local ethics committee (EK Nr 1306/2021) and registered (NCT06069752).

On the day of preoperative examination, all patients underwent a full ophthalmic examination, including slit lamp biomicroscopy. Routine optical biometry was performed (IOLMaster 700, Carl Zeiss Meditec AG, Germany) and IOL power calculation was performed using the Barrett Universal II formula in all cases, but for safety reasons, results between 6 different formulae were compared (Barrett Universal II, Haigis, SRK/T, HofferQ, Kane and EVO). Target refraction depended on the patient’s demand and ranged between emmetropia and − 2.50dpt. All patients underwent standard cataract surgery in topical anesthesia by one of four experienced surgeons (SM, NH, PL, RS). In all cases, both eyes were operated by the same surgeon. The order of conventional cataract surgery (CCS) and FLACS was randomly chosen using an online randomisation tool (randomizer.org), the second eye received the other treatment method. Pre-operative therapy was tropicamide 1% gtt, phenylephrine 2.5% gtt, and cyclopentolate 1% gtt.

In the CCS group, a self-sealing 2.4 mm clear corneal incision, injection of an ophthalmic viscoelastic device (OVD, Polyvisc 2.0%, Polytech Domilens GmbH, Germany), manual capsulorhexis with a size of 5.5 mm and phacoemulsification using a horizontal chop technique (OS4, Oertli, Switzerland) were performed. After irrigation/aspiration of cortical material and a refill of the capsular bag with OVD, an aspheric one piece IOL (CT LUCIA 621P, Carl Zeiss Meditec AG, Germany) was implanted in the capsular bag using the dedicated injector. Afterwards, the OVD was removed in front of and behind the IOL, wounds were hydrated, and cefuroxime was instilled in the anterior chamber in all cases.

In the FLACS group, treatment was started with the femtosecond laser (Victus, Bausch&Lomb, USA), which was used for a 5.00 mm capsulorhexis. Afterwards, the patient was positioned on the operating table in the same room, and the rest of the surgery was performed manually. After performing a 2.4 mm clear corneal incision and two paracenteses the anterior chamber was filled with the same OVD as in the conventional group. After reassuring that the anterior capsulotomy was complete, the rhexis flap was removed with the rhexis forceps. After mild hydrodissection and removing the gas bubbles phacoemulsification was performed. The phacoemulsification device and intraocular lens used were the same as for the CCS group.

Post-operative standard medication within the first month was Bromfenac gtt (Bausch&Lomb, USA) twice daily, and Tobradex eye drops (Novartis, Switzerland) 6 times daily for one week in all cases in both groups.

One, 6 and 12-months after surgery, slit-lamp examination was performed. Examination included IOL status regarding tilt, decentration, dislocation and glistening as well as location, intensity and clinical significance of posterior capsule opacification (PCO). Glistening was evaluated within a 10.0 mm x 2.0 mm field, using a scale ranging from 0 (none) to 4 (≥ 40), in increments of 10. PCO intensity grading was defined as none, mild, moderate, advanced and substantial loss of transparency in the posterior capsule. Significance was graded as non-significant, significant and significant requiring Nd: YAG.

Furthermore, optical coherence tomography (OCT) measurements (IOLMaster 700, Carl Zeiss Meditec AG, Germany, and CASIA 2, Tomey, Japan) as well as subjective refraction using the Jackson cross-cylinder method and ETDRS charts at 5 m (Precision Vision, USA) were performed at every follow-up visit. Contrast sensitivity was evaluated using monocular Pelli-Robson test under photopic conditions.

A Halo and Glare simulator (Eyeland Design Network GmbH, Germany, https://mediawebtool.com/counselor/vs.php?cnr=00VS-DEMO&mod=hal) was used to categorize the patients’ binocular photopic phenomena 6 and 12 months postoperatively. Patients were given the possibility to choose between three different forms of halo and glare, then the intensity of halo and glare phenomena were each adjusted on a scale of 0 to 100. After 12 months, patients were given a Catquest-9SF [3] and a QOV questionnaire [4]. 

The used hydrophobic acrylic monofocal one piece IOL (CT LUCIA 621 P) features a fragment of heparin in its surface coating, a modified C-loop haptic design, a 360° square edge optic step-vaulted design with an aspheric optic [5]. 

### Image analysis and statistical analysis

Data was analyzed using Microsoft Excel (v.16.12, Microsoft Corp, USA) and SPSS software (SPSSV 24.0; IBM, USA).

Descriptive analysis was performed, and results are presented as mean, standard deviation, and range. Data was tested for normal distribution using the Shapiro-Wilk test. A p-value below 0.05 was considered statistically significant. For comparison of measurements at 1, 6 and 12 months, Friedman’s test was used for non-normally distributed data.

## Results

A total of 100 eyes of 50 patients were included in this study, 54% female and 46% male. Mean age at surgery was 70.5 ± 7.2 years. Thirteen patients had to be excluded, 7 patients due to technical problems of the femtosecond laser (prior to start of the treatment), one patient due to intraoperative zonulolysis and 5 patients due to loss to follow-up. Overall, mean axial eye length was 23.26 ± 0.84 mm, mean ACD was 3.00 ± 0.36 mm, with no statistically significant differences between the two groups.

### Visual acuity and spherical equivalent

At 12 months, BCDVA was − 0.08 ± 0.07 logMAR and − 0.08 ± 0.08 logMAR for the CCS and the FLACS group, respectively. UDVA was 0.06 ± 0.11 logMAR and 0.05 ± 0.11 logMAR respectively. There was no statistically significant difference between both groups (BCDVA *p* = 0.989; UDVA = 0.472).

**Fig. 1:**
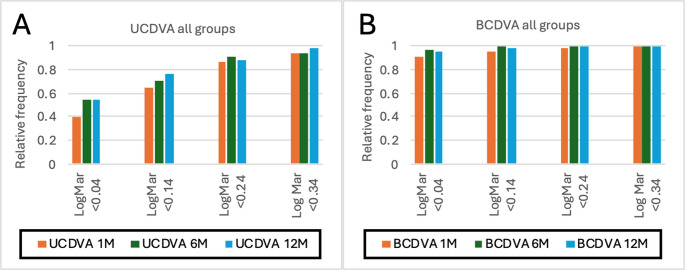
**A** and **B** Relative frequency of eyes within an uncorrected (UCVA) (1 A) and best corrected (BCDVA) (1B) distance visual acuity of < 0.04, 0.14, 0.24 and 0.34 logMar for both groups

Specifically, monocular visual acuity development for both groups over the course of 12 months is displayed in Fig. 1a and b. At 12 months, 94.59% of all eyes were within a UDVA < 0.24 logMar, with 85.14% demonstrating a UDVA of less than 0.14 logMAR.

At 12 months, the postoperative spherical equivalent (SEQ) was 0.23 ± 0.38 dpt in the CCS and 0.21 ± 0.46 dpt in the FLACS group, respectively, with no statistically significant difference between the groups (*p* = 0.180). Overall, SEQ at 12 months was 0.24 ± 0.42 dpt, with 79.73% of eyes within a spherical equivalent of ± 0.5dpt and 98.65% of eyes within a spherical equivalent of ± 1.0dpt.

**Fig. 2:**
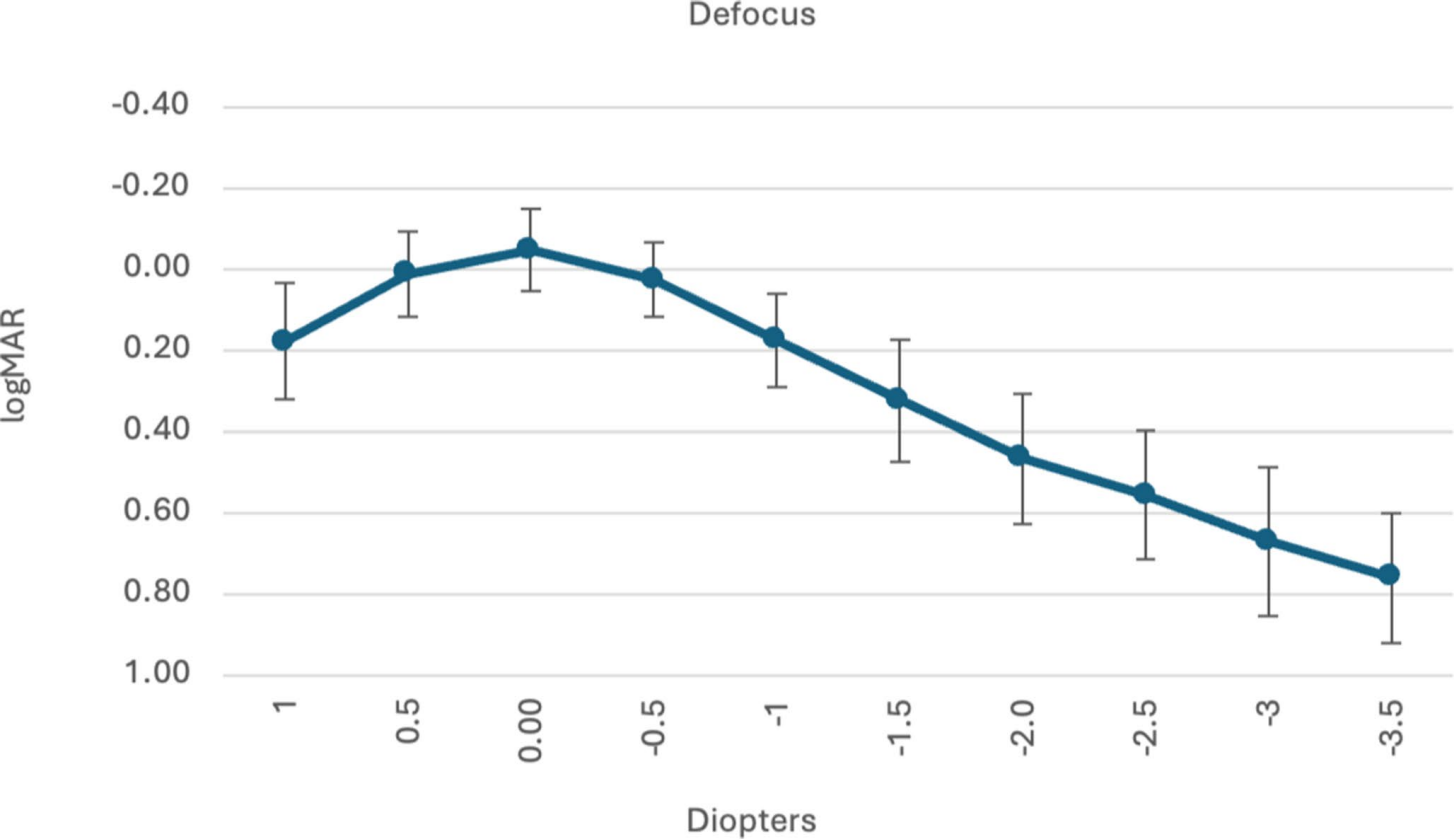
Monofocal defocus curve for both groups at 12 months

The monocular defocus curve is displayed in Fig. 2, depicting a mean visual acuity < 0.20 dpt within a range of + 1.0 to −1.0dpt.

### Refractive error

Mean refractive prediction error (ME) and mean absolute refractive prediction error (MAE) for all eyes were found to be 0.41 ± 0.44 dpt (median 0.36 dpt; range − 0.41 to 1.85 dpt) and 0.48 ± 0.38 dpt (median 0.39 dpt; range 0.01 to 1.85 dpt), respectively. No significant differences were observed in ME and MAE between the groups (*p* = 0.559 and *p* = 0.786, respectively). In the FLACS group, 64.86% of eyes were within 0.5 dpt, 78.38% within 0.75 dpt, 89.19% within 1.0 dpt, 97.30% within 1.5 dpt and 100.00% within 2.0 dpt. In the CCS group, 67.57% of eyes were within 0.5 dpt, 78.38% within 0.75 dpt, 86.49% within 1.0 dpt, and 100.00% within 1.5dpt.

### PCO + glistening

At month 12, mild posterior capsule opacification was present in 15 eyes (40.54%) in the CCS group and 14 (37.84%) eyes in the FLACS group (*p* = 1.000). In the CCS group the distribution of PCO was 31.25% peripheral, 12.50% paracentral and 56.25% diffuse compared to 28.57%, 7.14% and 64.29% in the FLACS group. PCO was considered not clinically significant in all cases as no vision deterioration occurred.

Two pairs of eyes (10.81%) had undergone a Nd: YAG capsulotomy at the six-month mark due to a significant progression of PCO. One eye in the CCS group exhibited anterior capsule phimosis. Glistening was present in one pair of eyes with only mild glistening, corresponding to an amount < 10 in a 10 × 20 mm field.

There were no other relevant slit lamp findings.

### Contrast sensitivity

With regard to monocular contrast sensitivity, mean logCS for the FLACS group at 12 months was 1.51 ± 0.12, compared to 1.52 ± 0.13 for the CCS group. No significant difference was observed between the two groups (*p* = 0.331).

### Halo + glare

Subjectively, eleven patients (29.73%) experienced binocular optical phenomena at 12 months. Seven patients (18.92%) experienced glare, with a mean size of 2.03 ± 5.18 and a mean intensity of 5.78 ± 17.90. Eleven patients (29.73%) experienced halos, subdivided in type 1 (9 patients, 24.32%) and type 2 halos (2 patients, 5.40%) with a mean size of 4.25 ± 7.45 and a mean intensity of 9.66 ± 16.40. One patient experienced both halo and glare with an intensity > 50 (Glare size 21, intensity 95, Halo type 2, size 12, intensity 58).

### Cat quest 9SF + QOV

The CatQuest 9-SF and QOV outcomes are displayed in Tables 1 and 2. In 83.78% of patients, there were no difficulties with daily activities. The largest difficulty was found when reading text in newspapers, with 33.43% of patients experiencing at least some difficulty. Overall, 97.30% of all patients were very or fairly satisfied with the postoperative outcome, with only one patient being dissatisfied.

**Table 1:** Postoperative data on capsular stability as well as the shift of anterior chamber depth (ACD), Tilt and decentration between 1 month to 6 months and 6 months to 12 months for both subgroups. CCS = conventional cataract surgery, flacs = femtolaser assisted cataract surgery

Cat-Quest-9SF	**Yes, very great difficulty**	**Yes, great difficulty**	**Yes, some difficulty**	**No, no difficulty**
Do you find that your sight at present in some way causes you difficulty in your everyday life?	0.00	2.70	13.51	83.78
	**Very dissatisfied**	**Fairly dissatisfied**	**Fairly satisfied**	**Very satisfied**
Are you satisfied or dissatisfied with your sight at present?	0.00	2.70	32.43	64.86
Do you have difficulty with the following activities because of your sight:	**Yes**,** very great difficulty**	**Yes**,** great difficulty**	**Yes**,** some difficulty**	**No**,** no difficulty**
Reading text in newspapers	0.00	2.70	29.73	67.57
Recognizing the faces of people you meet	0.00	2.70	13.51	83.78
Seeing the prices of goods when shopping	0.00	0.00	21.62	78.38
Seeing to walk on uneven surfaces, e.g. cobblestones	0.00	0.00	21.62	78.38
Seeing to do handicrafts, woodwork etc.	0.00	0.00	24.32	70.27
Reading subtitles on TV	0.00	0.00	8.11	91.89
Seeing to engage in an activity/hobby that you are interested in	0.00	0.00	8.11	91.89

**Table 2:** The results of the Catquest 9-SF questionnaire are presented as percentages of patients who selected the respective responses

QOV	Never	Occasionally	Quite often	Very often
How often do you experience glare?	52.63	44.74	0.00	0.00
How often do you experience haloes?	81.08	16.22	0.00	2.70
How often do you experience starbursts?	70.27	29.73	0.00	0.00
How often do you experience hazy vision?	83.78	16.22	0.00	0.00
How often do you experience blurred vision?	78.38	21.62	0.00	0.00
How often do you experience distortion?	100.00	0.00	0.00	0.00
How often do you experience a fluctuation in your vision?	56.76	43.24	0.00	0.00
How often do you experience focusing difficulties?	72.97	27.03	0.00	0.00
How often do you experience difficulty judging distance or depth perception?	86.49	13.51	0.00	0.00
	**Not at all**	**Mild**	**Moderate**	**Severe**
How severe is the glare?	54.05	37.84	8.11	0.00
How severe are the haloes?	78.38	18.92	2.70	0.00
How severe are the starbursts?	67.57	27.03	5.41	5.41
How severe is the hazy vision?	83.78	13.51	2.70	0.00
How severe is the blurred vision?	83.78	16.22	0.00	0.00
How severe is the distortion?	100.00	0.00	0.00	0.00
How severe are the double or multiple images?	91.89	2.70	5.41	0.00
How severe is the fluctuation in your vision?	62.16	37.84	0.00	0.00
How severe are the focusing difficulties?	78.38	18.92	2.70	0.00
How severe is the difficulty judging distance or depth perception?	86.49	13.51	0.00	0.00
	**Not at all**	**A little**	**Quite**	**Very**
How bothersome is the glare?	64.86	35.14	0.00	0.00
How bothersome are the haloes?	86.49	10.81	2.70	0.00
How bothersome are the starbursts?	78.38	21.62	0.00	0.00
How bothersome is the hazy vision?	86.49	13.51	0.00	0.00
How bothersome is the blurred vision?	89.19	10.81	0.00	0.00
How bothersome is the distortion?	100.00	0.00	0.00	0.00
How bothersome are the double or multiple images?	94.59	5.41	0.00	0.00
How bothersome is the fluctuation in your vision?	70.27	27.03	0.00	0.00
How bothersome are the focusing difficulties?	81.08	18.92	0.00	0.00
How bothersome is the difficulty judging distance or depth perception?	86.49	13.51	0.00	0.00

### Tilt

Tilt was evaluated at 1, 6 and 12 months for both groups individually as well as for the entire patient cohort. Specifications for each group as well as the postoperative shift between 1 and 6 months and 6–12 months are displayed in Table 3.

**Table 3:** The results of the quality of vision (QOV) questionnaire are presented as percentages of patients who selected the respective responses

		Group	Mean	SD	Median	Min	Max
ACD (mm)	1 M	FLACS	4.63	0.23	4.62	4.23	5.18
		CCS	4.65	0.25	4.61	4.24	5.38
		Total	4.64	0.24	4.61	4.23	5.38
	6 M	FLACS	4.61	0.26	4.63	4.22	5.19
		CCS	4.61	0.27	4.59	4.12	5.23
		Total	4.61	0.26	4.62	4.12	5.23
	12 M	FLACS	4.64	0.26	4.60	4.10	5.24
		CCS	4.60	0.22	4.57	4.19	5.15
		Total	4.62	0.24	4.58	4.10	5.24
ACD Shift (mm)	1-6 M	FLACS	0.12	0.12	0.06	0.01	0.47
		CCS	0.28	0.17	0.31	0.01	0.69
	6-12 M	FLACS	0.10	0.08	0.08	0.00	0.40
		CCS	0.17	0.25	0.07	0.00	1.03
Tilt (°)	1 M	FLACS	5.15	1.71	5.20	1.40	8.70
		CCS	4.90	1.80	4.67	1.90	10.50
		Total	5.03	1.75	4.90	1.40	10.50
	6 M	FLACS	4.82	2.22	4.60	0.80	10.80
		CCS	4.31	1.65	4.20	0.20	7.60
		Total	4.57	1.96	4.50	0.20	10.80
	12 M	FLACS	4.69	2.17	4.80	1.00	11.70
		CCS	4.99	2.78	4.80	0.14	13.60
		Total	4.84	2.48	4.80	0.14	13.60
Tilt Shift (°)	1-6 M	FLACS	1.40	1.09	1.20	0.00	3.60
		CCS	1.63	1.49	1.20	0.00	6.50
	6-12 M	FLACS	1.19	1.62	0.60	0.00	7.10
		CCS	1.78	1.71	1.40	0.00	6.90
Decentration (mm)	1 M	FLACS	0.24	0.15	0.22	0.03	0.74
		CCS	0.28	0.17	0.26	0.02	0.63
		Total	0.26	0.16	0.23	0.02	0.74
	6 M	FLACS	0.28	0.15	0.30	0.01	0.67
		CCS	0.34	0.20	0.33	0.04	0.77
		Total	0.31	0.18	0.31	0.01	0.77
	12 M	FLACS	0.54	0.69	0.32	0.06	1.07
		CCS	0.49	0.43	0.35	0.01	1.77
		Total	0.46	0.36	0.33	0.01	1.77
Decentration Shift (mm)	1-6 M	FLACS	0.14	0.13	0.09	0.00	0.64
		CCS	0.13	0.13	0.10	0.00	0.60
	6-12 M	FLACS	0.25	0.26	0.16	0.00	0.87
		CCS	0.32	0.38	0.20	0.00	1.60

Comparison of differences in tilt between visits both individually and overall showed no significant changes (FLACS: *p* = 0.132; CCS: *p* = 0.939; all: *p* = 0.433).

### Decentration

Evaluation of decentration at 1, 6 and 12 months was performed for both subgroups as well as overall. Decentration data as well as the postoperative shift between 1 and 6 months and 6–12 months is displayed in Table 3.

There were significant changes of decentration between visits in the FLACS subgroup (*p* = 0.036) as well as overall (*p* = 0.003).

Post hoc testing for the FLACS group showed a significant change between the 1 M and 12 M visit (*p* = 0.040). Overall, post hoc testing showed significant changes between the 1 month and 6 month as well as 1 month and 12 month visit (*p* = 0.027 and *p* = 0.004 respectively).

There were no significant changes in decentration in the CCS subgroup (*p* = 0.060).

### Anterior chamber depth

Specifications for ACD at 1, 6 and 12 months as well as the shift between 1 and 6 months and 6–12 months are depicted in Table 3.

No significant changes in ACD between visits were found for the CCS subgroup or overall (*p* = 0.704 and *p* = 0.143 respectively).

Changes were found in the FLACS subgroup (*p* = 0.027), with post hoc testing indicating a significant shift between the 6- and 12-month visit (*p* = 0.032).

## Discussion

This study evaluated the safety and efficacy of a new FDA approved monofocal IOL after CCS and FLACS.

In this study, the CT LUCIA 621 P showed very good refractive stability in the first year postoperatively with 79.73% of patients within 0.5 dpt and 98.65% within 1.00 dpt prediction error, which is similar to a study of Schallhorn et al. about the IOL’s predecessor CT LUCIA 611P, analyzing a total of 339 eyes [6]. In a prospective study using the same IOL model with a focus on patients with pseudoexfoliation syndrome, Borkenstein and Borkenstein reported that all patients’ refraction fell within the 0.5 dpt range [7]. However, a retrospective analysis of the CT LUCIA 621P, using a different biometer and different formulae, reported that only 68% of their patients’ refraction fell within 0.5 dpt [8]. 

In our study we observed a mild postoperative hyperopic shift with a mean SEQ of 0.24 ± 0.41 dpt. The Barrett Universal II formula was used for IOL power calculation in all cases. This shift regarding the target refraction was also observed with respect to ME and MAE, with 64.86% of all eyes within a refractive prediction error of ± 0.5 dpt, a percentage lower than that reported by Lundström et al. in the 2019 EUREQUO report [9]. 

There was also no significant difference in ME and MAE between groups. Again, we could not find an explanation for this deviation, as our study population was homogenous regarding preoperative biometric data. However, an optimization of the IOL power calculation formula constant should be considered.

In the current study, we found a proportion of 2 patients requiring Nd: YAG capsulotomy within the first 12 months postoperatively. PCO development is multifactorial, however the IOL design plays a major role in the PCO protection [10] and the development of sharp-edged optics has greatly reduced the PCO incidence [11]. Therefore, the square edged 360-degree optic which is featured by the CT LUCIA 621P is believed to be one of the inhibitory factors of PCO by providing a mechanical barrier for lens epithelial cell migration [12]. 

Comparing rates of PCO requiring Nd: YAG capsulotomy is however difficult, as it highly depends on the judgment of the PCO severity by the examiner rather than on the IOL material itself. Additionally, time has to be taken into account, as a study by Duman et al. found that the most Nd: YAG capsulotomies are performed 3 years after cataract surgery [13]. This current study observed patients only 12 months postoperatively.

IOLs made of hydrophobic acrylic material often are described to feature a phenomenon called glistening. However, the susceptibility to glistening varies between different IOL manufacturers [14]. Although the impact of IOL glistening on BCVA and visual quality is controversially discussed, the efforts to develop glistening free biomaterials are ongoing [15]. In this study we could only detect mild glistening in one pair of eyes during the postoperative 12 month period.

With regards to visual acuity, we found satisfactory postoperative outcomes with more than 98% of patients reporting a BCDVA and UDVA ≤ 0.10 logMAR. That corresponds to a reported 97.30% satisfactory rate in our patients according to the CatQuest-9SF.

One patient of our study cohort was fairly dissatisfied with her sight at present. When looking at the related data, we found that the patient in question also had the highest intensity of halo and glare, however at a UDVA of 0.0 logMAR. This patient showed no signs of PCO and had no other IOL related findings.

In the current study we found a significant change in decentration within visits. However, those patients affected by an increased decentration did not report any visual disturbances, which might be due to the IOLs Zeiss optic asphericity design, reducing the IOL’s sensitivity to misalignment, as shown by Yan et al. in an optical bench study. Due to its step vaulted haptic-optic design, the IOL itself is less inflicted by decentration or tilt [7]. A gradually increasing decentration might however be caused by capsular shrinkage postoperatively [16]. 

To summarize, this study aimed to evaluate the refractive predictability and positional stability of a new IOL in the capsular bag. The investigated IOL was found to be safe and stable.
