# Correction: Capsular bag performance of an aspheric hydrophobic IOL after conventional phacoemulsification or femtosecond laser-assisted cataract surgery: 12-month results of a randomized prospective study

**DOI:** 10.1007/s00417-025-07045-8

**Published:** 2025-11-29

**Authors:** Marina Casazza, Sophia Anna Reifeltshammer, Nino Hirnschall, Jascha Wendelstein, Siegfried Mariacher, Peter Laubichler, René Siska, Matthias Bolz

**Affiliations:** 1 Department of Ophthalmology, Medical University Graz, Neue Stiftingtalstraße 6, 8010 Graz, Austria; 2 Department of Ophthalmology and Optometry, Kepler University Hospital, Linz, Austria; 3 Medical Faculty, Johannes Kepler University, Linz, Austria; 4 Department of Ophthalmology, Ludwig-Maximilians-University, Munich, Germany; 5 Private Practice, Landeck and Innsbruck, Austria

**Correction: Graefe's Archive for Clinical and Experimental Ophthalmology (2025) 263:2817-2825**

10.1007/s00417-025-06919-1

In the Results section of the originally published article, the following sentence contained an incorrect percentage:

“Two pairs of eyes (10.81%) had undergone a Nd:YAG capsulotomy at the six-month mark due to a significant progression of PCO.”

The correct version is:

“Two pairs of eyes (5.41%) had undergone a Nd:YAG capsulotomy at the six-month mark due to a significant progression of PCO.”

The authors regret this error and apologize for any inconvenience caused.

